# Novel PI3K/Akt Inhibitors Screened by the Cytoprotective Function of Human Immunodeficiency Virus Type 1 Tat

**DOI:** 10.1371/journal.pone.0021781

**Published:** 2011-07-12

**Authors:** Yuri Kim, Joseph A. Hollenbaugh, Dong-Hyun Kim, Baek Kim

**Affiliations:** 1 Department of Pharmacy, Kyung-Hee University, Seoul, South Korea; 2 Department of Microbiology and Immunology, University of Rochester Medical Center Rochester, Rochester, New York, United States of America; University of Hong Kong, Hong Kong

## Abstract

The PI3K/Akt pathway regulates various stress-related cellular responses such as cell survival, cell proliferation, metabolism and protein synthesis. Many cancer cell types display the activation of this pathway, and compounds inhibiting this cell survival pathway have been extensively evaluated as anti-cancer agents. In addition to cancers, several human viruses, such as HTLV, HPV, HCV and HIV-1, also modulate this pathway, presumably in order to extend the life span of the infected target cells for productive viral replication. The expression of HIV-1 Tat protein exhibited the cytoprotective effect in macrophages and a human microglial cell line by inhibiting the negative regulator of this pathway, PTEN. This cytoprotective effect of HIV-1 appears to contribute to the long-term survival and persistent HIV-1 production in human macrophage reservoirs. In this study we exploited the PI3K/Akt dependent cytoprotective effect of Tat-expressing CHME5 cells. We screened a collection of compounds known to modulate inflammation, and identified three novel compounds: Lancemaside A, Compound K and Arctigenin that abolished the cytoprotective phenotype of Tat-expressing CHME5 cells. All three compounds antagonized the kinase activity of Akt. Further detailed signaling studies revealed that each of these three compounds targeted different steps of the PI3K/Akt pathway. Arctigenin regulates the upstream PI3K enzyme from converting PIP2 to PIP3. Lancemaside A1 inhibited the movement of Akt to the plasma membrane, a critical step for Akt activation. Compound K inhibited Akt phosphorylation. This study supports that Tat-expressing CHME5 cells are an effective model system for screening novel PI3K/Akt inhibitors.

## Introduction

Viral infections alter numerous cellular signaling pathways. Among these pathways, the PI3K/Akt cell survival pathway is activated by several key human pathogenic viruses such as human papillomavirus (HPV; [1]), hepatitis virus C (HCV; [2]), human T cell leukemia virus (HTLV; [3]) and human immunodeficiency virus Type 1 (HIV-1; [4], [5], [6], [7], [8]). This virus-induced activation of PI3K/Akt pathway involves specific viral proteins such as E6/E7 of HPV, NS5A of HCV, Tax of HTLV and Tat of HIV-1 [1], [3], [4], [9]. Interestingly, in contrast to other viral proteins that activate the PI3K/Akt pathway, the expression of Tat appears to inactivate Phosphatase and tensin homolog (PTEN), the negative regulator of PI3K/Akt pathway [8], [10]. The genetic inactivation of PTEN is also closely tied to the development of human cancers [11], [12]. Cell transformation is the consequence of direct PI3K/Akt activation by onco-viruses (i.e. HPV, HCV and HTLV) [1], [3], [9]; and indeed, the PI3K/Akt pathway is highly activated in many cancer cell types [13]. Many pharmacological PI3K/Akt inhibitors have been extensively evaluated as potential anti-cancer agents, which can abolish the capability of cancer cells to extend their life span against anti-cancer pressures such as cellular immune response and inflammation. Structure-based drug design against various cellular kinases involved in the PI3K/Akt pathway has been extensively used to search for anti-PI3K/Akt agents [14]. Recently, a non-human cell line that overexpressed human Akt kinase was also used for screening of Akt inhibitors [15]. Even with these broad efforts, PI3K/Akt inhibitors that are safe and effective for clinical use remain limited.

The expression of HIV-1 Tat protein in the human microglial CHME5 cell line, as well as human primary macrophages, activates the PI3K/Akt pathway upon exposure to cellular stresses by reducing the level of PTEN, rendering a strong resistance to extracellular stresses such as LPS or nitric oxide [7], [8], [16]. We believe that this enhanced cell survival phenotype of Tat-expressing human microglia and macrophages plays an important role for the establishment of long-lived HIV-1 reservoirs in the CNS [17], [18], [19], [20], which in turn induces neuronal death and HIV-1 associated neurodegenerative diseases [21], [22].

In this report, we employed the Tat-induced cytoprotective phenotype of CHME5 cells for screening of anti-PI3K/Akt agents from a collection of chemical compounds known to modulate cellular inflammation, which is one of the key cellular stresses associated with viral infection and pathogenesis. We identified three compounds: Lancemaside A1 (LA), Compound K (CK) and Arctigenin (AR) that effectively abolished the Tat-induced cytoprotective phenotype of Tat-expressing CHME5 cells. Importantly, these three compounds negatively modulated three different steps of the PI3K/Akt cell survival network, PI3K, Akt activation and Akt kinase activity.

## Materials and Methods

### Reagents, cell lines and virus

The previously established CHME5 cell lines were used for this study [7]. In short, CHME5 cells were transfected with pcDNA3.1 hygromycin (control) or pcDNA-Tat101 (tat). Human lung fibroblasts (HLF; purchased from ATCC) were also used for this study, and the cells were maintained in 10% FBS in DMEM media. Adenoviruses: Ad-eGFP and Ad-Akt-PH-eGFP were previously established [7]. Antibodies used in these studies were purchased from Cell Signaling.

### Chemicals

Lipopolysaccharide (LPS; *E. coli* serotype O26:B6), cycloheximide (CHX), puerarin, apigenin, aucubin, quercetin and kaempherol, isophytolased were purchased from Sigma (St. Louis, MO, USA). Silybinin and silymarin were prepared according to previously reported methods [23]. Naringenin, nobiletin, tangeretin poncirin and naringin were isolated from the immaturus fructus of *Citrus unshiu* and *Poncirus trifoliate* according to previously reported methods [24]. Ginsenoside Rb1 and ginsenoside Rg3 were isolated from the rhizome of *Panax ginseng Meyer* according to the previously reported method [25]. Kalopanaxsaponin B was isolated from the stem bark of *Kalopanax pictus* according to the previously reported method [26]. Lancemasides were isolated from the rhizome of *Codonopsis lanceolata* according to the previously reported method [27]. Soyasaponin B was isolated from soybean according to the previously reported method [28]. Glycitein and tectoridin were isolated from the flos of *Pueraria thunbergiana* as previously described [29]. Compound K and Lancemaside A1 purifies were greater than 95%. Arctigenin purity was greater than 93%. The three lead compound chemicals were dissolved in DMSO.

### Screening protocol using the Tat-expressing CHME5 cell line

CHME5 subline cells expressing Tat protein were treated with 50 µg/ml LPS and 10 µg/ml CHX stress in the presence or absence of 10 µM drugs, and the live/dead assay (Invitrogen) was performed on CHME5 cells as per the manufacturer's protocol. Cell images were captured with a fluorescence microscope and then dead cells (red) and live cells (green) were counted manually. A representative data set is shown in Data S1. Data S2 shows the cell death for CHME5 control cells treated under different drug conditions.

### Western blot analysis

For monitoring the changes in Akt, pAkt, GSK3b and pGSK3b levels, Tat-expressing CHME5 cell subline was exposed to LPS/CHX in the presence or absence of three compounds for 90 minutes. The levels of total Akt and pAkt proteins were determined and used as a ratio to normalize the data. Complete data set for mean and standard deviations for the western blots is shown in Supplementary Table S1. For PDK1, cells were treated with the three compounds (20 mM) with and without LPS/CHX stress for 20 minutes. The level of pPDK1 was normalized to β-actin and labeled below the bands. The cell culture conditions for each western analysis are described in the figure legends.

### Akt membrane migration

One million Tat-expressing CHME5 cells were pre-treated with the three compounds (20 µM) for 20 minutes. Then cells were treated with and without LPS/CHX stress for 60 minutes. Lysates were prepared from an equal number of the treated cells using a compartmental protein extraction kit (Millipore). The membrane extract lysates were analyzed by western dot blot (Echelon) and visualized with anti-PIP3 antibody. A standard curved using known amounts of PIP3 was generated (Data S3) and then used to determine the quantity of PIP3 in the treated cell lysates. The band densities were calculated and normalized by PTEN protein, used as an internal loading control. For Adenovirus studies, HLF cells were pre-treated with the three compounds (20 µM) for 20 minutes before incubation with 100 nM EGF for 40 minutes. Akt plasma membrane localization was visualized by the movement of Akt-PH-eGFP using a fluorescence microscope (Zeiss).

## Results

### Tat-induced cytoprotective phenotype as a screening tool for anti-PI3K/Akt agents

**Figure 1:**
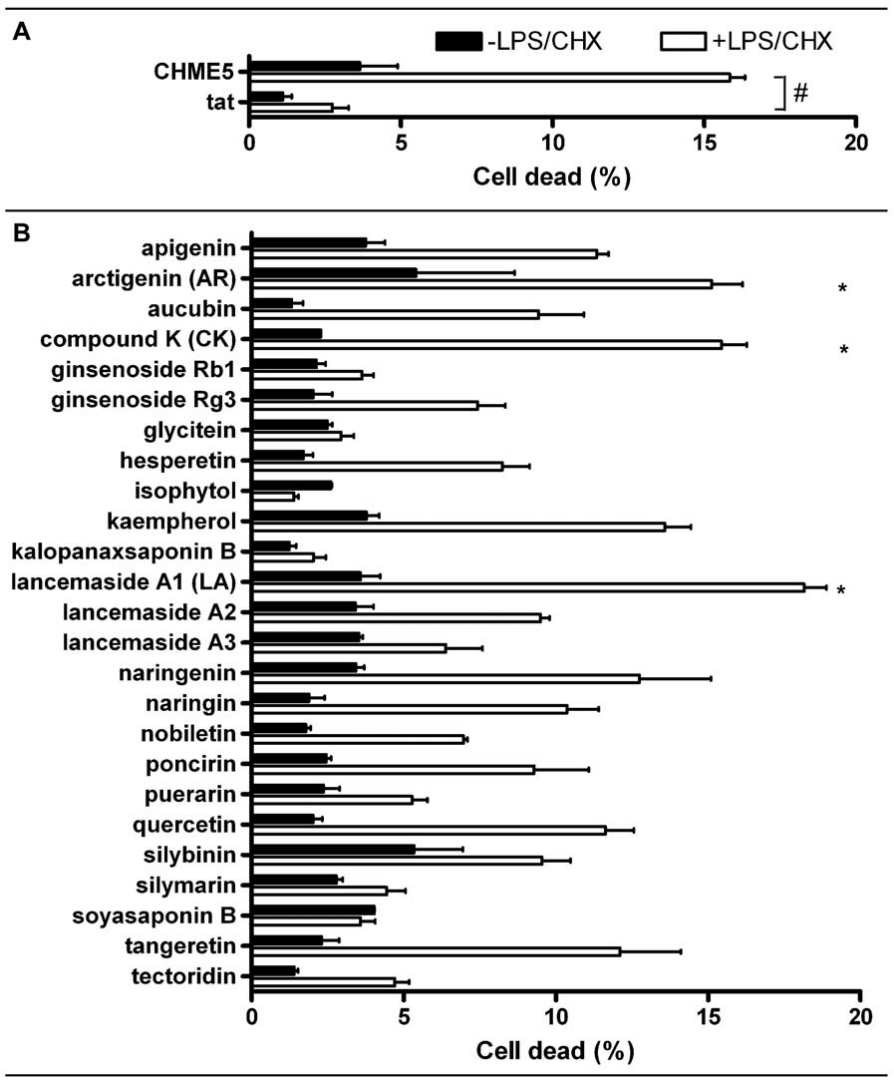
Compounds screened for inhibiting cytoprotective effect. (**A**) CHME5 control cells and Tat-expressing CHME5 cells were treated with (open bars) and without (closed bars) 50 µg/ml LPS and 10 µg/ml cycloheximide (LPS/CHX) to show the baseline level of death due to external stress (∼3%). Induction of cell death by LPS/CHX stress is denoted by “#” for the CHME5 control cells. (**B**) The Tat-expressing CHME5 cells were treated with 25 different drugs (10 µM) in the presence or absence of LPS/CHX for 24 hours. Cell death was determined using the Live/Dead assay (Invitrogen). Arctigenin, Compound K and Lancemaside A1 were the most potent compounds that prevented the cytoprotective effect of Tat during LPS/CHX stress and are marked with asterisks (*). Data are plotted mean and SEM for 2–3 independent experiments for each drug.

We previously reported that the expression of HIV-1 Tat protein elevates the cytoprotective phenotype of human primary macrophages and a human microglial cell line, CHME5 [7], [8]. To monitor the cytoprotective phenotype of the Tat-expressing CHME5 cells, we determined cell death by imaging cells for fluorescence using ethidium homodimer for dead cells (red) and calcein for live cells (green) (Data S1). Data from three experiments were compiled and are shown in Figure 1A. CHME5 control cells (control) stably transfected with pcDNA3.1-hygromycin exhibit ∼16% cell death upon the co-treatment with lipopolysaccharide (LPS, 50 µg/ml) and cycloheximide (CHX, 10 µg/ml), while the Tat-expressing CHME5 cells (tat) show a low baseline cell death (<3%) even in the presence of the LPS/CHX treatment. Both control and Tat-expressing CHME5 cell lines displayed 4% or less death when cultured without LPS/CHX.

### Identifying compounds that show anti-cytoprotective effects in Tat expressing CHME5 cells

**Figure 2:**
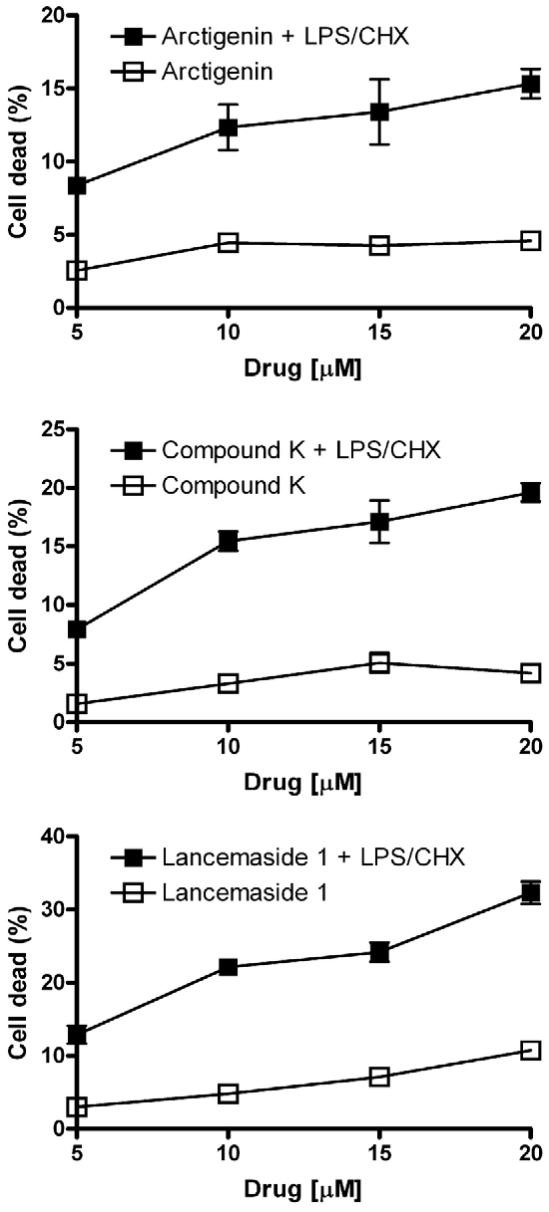
Dose dependent cell death curves of Tat-expressing CHME5 cells. The Tat-expressing CHME5 cell subline was incubated with four concentrations (5, 10, 15 and 20 µM) of Arctigenin, Compound K and Lancemaside A1 in the presence (black square) and absence (open square) of LPS/CHX stress. Cell death was determined using the Live/Dead assay and plotted as mean with SEM. See Data S1 for the live and dead cell staining of the Tat-expressing CHME5 cells with the three compounds at 10 µM.

Twenty-five compounds were previously isolated from parental chemical libraries and selected for their ability to modulate inflammatory stress responses. We investigated if these compounds were able to inhibit the cytoprotective phenotype in Tat-expressing CHME5 cells. As shown in Figure 1B (see “*”), among the 25 compounds tested at 10 µM, three compounds: Lancemaside A1 (LA), Arctigenin (AR) and Compound K (CK) induced cell death upon exposure to LPS/CHX. Importantly, cells treated with Lancemaside A1 and Compound K had similar cell death as compared to control (empty vector) CHME5 cell line in the absence of the LPS/CHX stress (Figure 1A) at the tested concentrations, whereas cells treated with Arctigenin alone showed roughly 6% cell death. These data led us to select the top three compounds that abolish the Tat-induced cytoprotective phenotype of CHME5 cells presumably by antagonizing PI3K/Akt pathway, which is the mechanism that elevates the survival capability of the Tat-expressing CHME5 [7], [16]. Next, dose concentration curves (5, 10, 15 and 20 µM) for the three drugs were determined. As shown in Figure 2, with an increase in drug concentration, a higher percentage of CHME5 cell death was detected in the LPS/CHX treatment groups. However, these drugs alone induce only base-line cell death (∼5%), indicating the absence of the cytotoxicity of these three compounds at the tested concentrations.

### Determining the effects of the screened compounds on Akt activation and kinase activity

**Figure 3:**
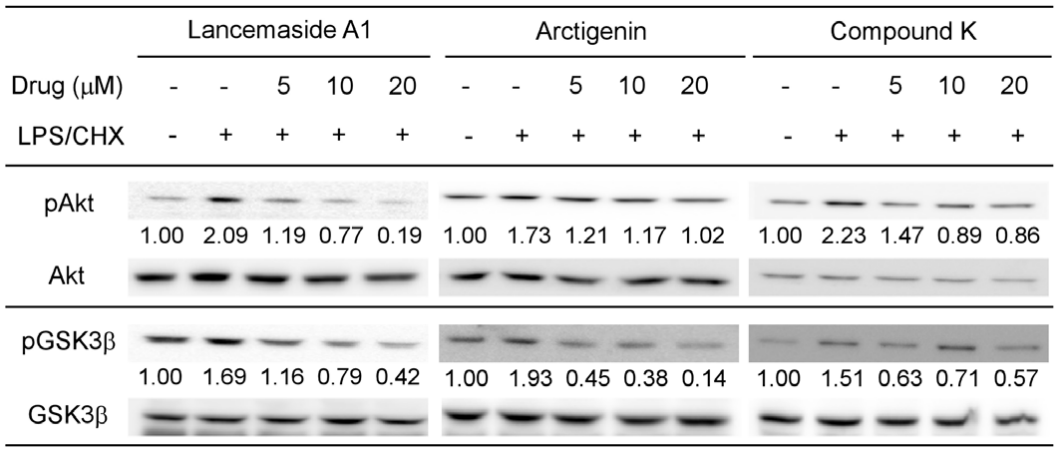
Examining the effects of the screened compounds on phosphorylation of Akt and GSK3β. Western blot analysis was performed on Tat-expressing CHME5 cell lysates treated with and without LPS/CHX in the presence of the three different drug compounds at 5, 10, and 20 µM concentrations for 90 minutes. Blots were probed for antibodies against phospho-Akt (pAkt), total Akt (Akt), phospho-GSK3β (pGSK3β), total GSK3β (GSK3β) and β-actin. The changes in amount of pAkt and pGSK3β levels were determined, and the mean values of the duplicates were indicated below each blot. The control lane, no LPS/CHX and drug, were set to 1. Data are representative of two independent experiments.

Next, we tested if Lancemaside A1, Arctigenin and Compound K can modulate the activation of Akt kinase, because Akt is a key molecule for PI3K/Akt survival pathway. Western blot analysis was used to monitor the phosphorylation of Akt kinase at three different drug concentrations (5, 10, 20 µM, Figure 3). Tat-expressing CHME5 cells were treated in the presence or absence of these compounds and LPS/CHX stress for 90 minutes. Lysates were than prepared and analyzed by western blot analysis to examine the levels of total Akt, phospho-specific Akt (pAKT, S473) and β-actin, used as a loading control.

As shown in Figure 3, the Tat-expressing CHME5 cells exhibited low levels of Akt phosphorylation (pAkt) without any stress (control lanes), whereas cells treated with LPS/CHX stress significantly induced the phosphorylation of Akt, which explains the strong survival phenotype. However, Tat-expressing CHME5 cells treated with the three different compounds have decreased levels of pAkt in a dose dependent manner (Figure 3). The relative density of each pAkt band was normalized with the total Akt level for each condition and then the fold changes in pAkt levels as compared to the LPS/CHX treatment groups were calculated. The averages of two experiments are shown below the western blot for each of the three compounds (Figure 3). All three compounds, particularly Lancemaside A1, effectively blocked the LPS/CHX stress induced pAkt in the Tat-expressing CHME5 cells, which explains the compound-induced loss of cell survival phonotype upon LPS/CHX treatment (Figure 2). Next, we monitored by western blot analysis the phosphorylated form of GSK3β (pGSK3β), total GSK3β, and β-actin. GSK3β is a substrate of Akt kinase and is inactivated upon phosphorylation. Similarly, the fold change of the normalized pGSK3β level upon the LPS/CHX treatment was used to compare the screened compounds as described above for the Akt phosphorylation. As shown in Figure 3, the levels of pGSK3β were decreased correspondingly with increasing concentrations of the three compounds. Collectively these data support that the cell death phenotype (Figure 1), which was first used to screen these compounds, is Akt dependent and that the three compounds are involved in reducing the pAkt level in the cell.

### Evaluating the effects of the screened compounds on PI3K activity and PTEN

**Figure 4:**
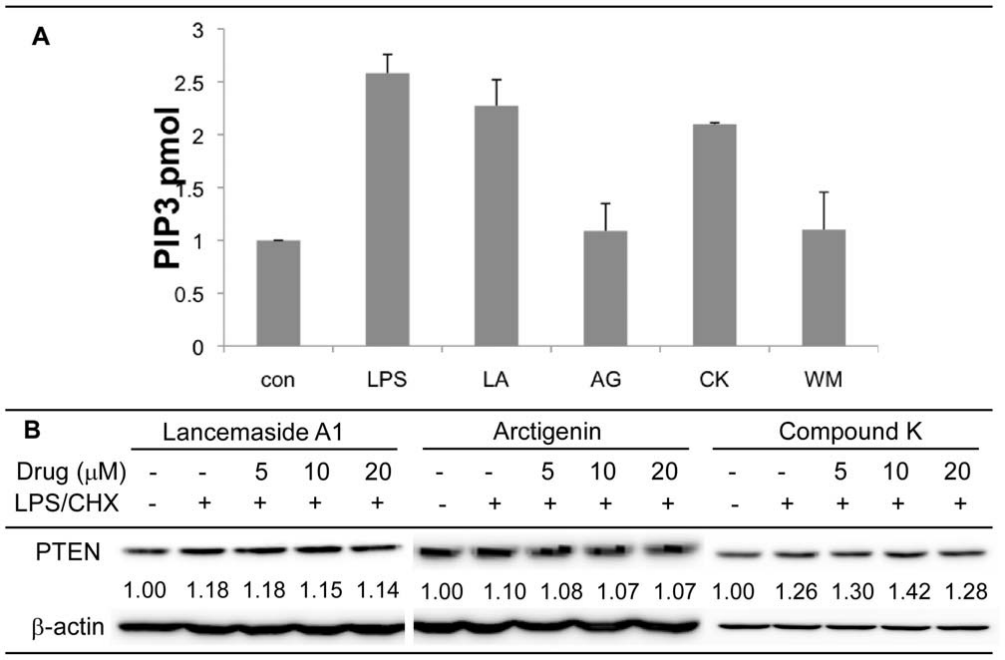
Determining the effects of three screened compounds on membrane PIP3 and PTEN levels. (**A**) Tat-expressing CHME5 cells were treated with and without LPS/CHX in the presence of the three different drug compounds at 20 µM for 20 minutes. Cell lysates were prepared for determining the membrane PIP3 level. Data were calculated by generating a standard curve of known concentrations for PIP3 provided from the manufacturer (Data S2). Control (Con), no treatment; LPS, 50 µg/ml LPS and 10 µg/ml cycloheximide; Lancemaside A1 (LA), Arctigenin (AR), Compound K (CK) and Wortmannin (WM). (**B**) Tat-expressing CHME5 cells were treated in the presence or absence of LPS/CHX and 5, 10, and 20 µM of each drug for 20 minutes. Cellular membrane fractions were processed and examined by western blot analysis for PTEN. β-actin was used as a loading control. The fold changes in the PTEN level were determined as described in methods.

While Lancemaside A1, Arctigenin and Compound K reduced Akt kinase activity and phosphorylation, it is possible that these three compounds might target different upstream regulation steps of the PI3K/Akt pathway. First, we investigated the effects of the screened compounds on PI3K activity by determining the level of PIP3 at the plasma membrane. This was done by employing a western dot blot-based assay and quantitating PIP3 level using a standard curve (Data S3). As shown in Figure 4A, the LPS/CHX treatment alone increased the level of PIP3, which is the key initial signature of PI3K pathway activation. Interestingly, Arctigenin reduced the level of PIP3 in the Tat-expressing CHME5 cells treated with the LPS/CHX stress, while neither Lancemaside A1 nor Compound K affected the levels of PIP3. Wortmannin is a well-known PI3K inhibitor and was used as a control to inhibit PIP3 induction in the presence of LPS/CHX (Figure 4A). These data suggest that unlike Lancemaside A1 and Compound K, Arctigenin likely targets the upstream PIP3 effector of the PI3K/Akt pathway.

Next, we examined if the screened compounds independently regulate the PTEN level within cells. PTEN serves as the key negative regulator of the PI3K/Akt pathway by converting PIP3 to PIP2. Arctigenin may be increasing the PTEN level, which results in the reduction of the PIP3 level observed in Figure 4A. Therefore, we tested if the screened compounds affect the expression level of PTEN in the Tat expressing CHME5 cells upon exposure to LPS/CHX stress. As shown in Figure 4B, none of the three screened compounds altered PTEN protein levels at the tested drug concentrations. Thus we conclude from these data that the PI3K/Akt pathway inhibition by the three compounds is PTEN independent. Importantly, since we did not observe an increase in PTEN levels during Arctigenin treatment, the reduction of PIP3 level by this compound is likely due to the direct inhibition of PI3K activity.

### Determining the effects of the screened compounds on PDK1

**Figure 5:**
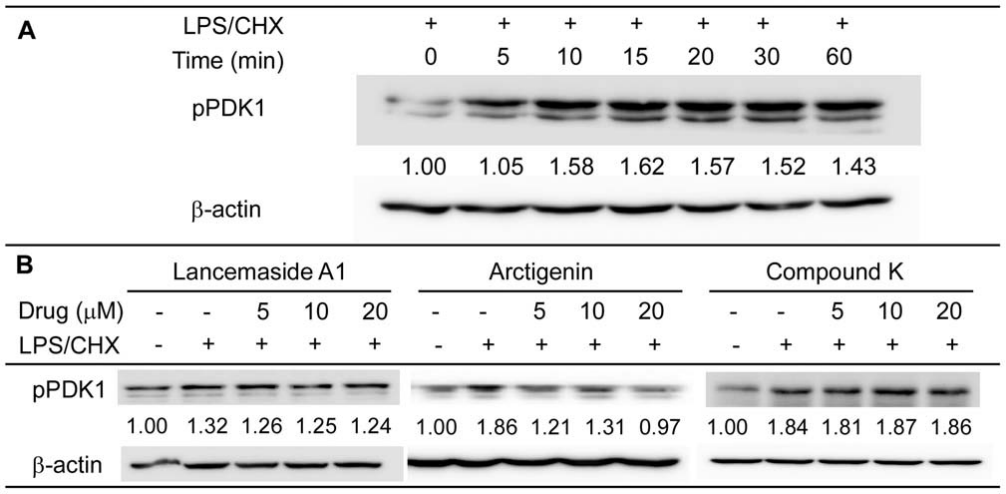
Examining the effects of the screened compounds on PDK1 phosphorylation. (**A**) A time course of PDK1 phosphorylation (pPDK) was performed to determine the optimal time point for maximum detection of pPDK1, which was achieved within 10 minutes of LPS/CHX treatment. (**B**) Cell lysates of Tat-expressing CHME5 cells treated for 20 minutes in the presence and absence of LPS/CHX. pPDK1 data were normalized to β-actin and the mean values are shown below the blot. Data are representative from two independent studies.

PDK1 is a direct downstream kinase of PI3K and activates the membrane bound Akt protein. First, we performed a time course to shown that LPS/CHX treatment of Tat-expressing CHME5 cells led to an increase in phospho-PDK1 (pPDK1) level. We found that the maximum pPDK-1 was obtained within 10 minutes of LPS/CHX treatment and was maintained for more than 60 minutes (Figure 5A). Next, we examined the effect of these three compounds on pPDK1 levels, which were evaluated 20 minutes after treatment. As shown in Figure 5B, neither Lancemaside A1 nor Compound K influenced the levels of pPDK1 induced by LPS/CHX treatment, while only Arctigenin reduced the levels of pPDK1 in a concentration dependent manner, reaching untreated control levels with 20 µM Arctigenin. These data suggest that both Lancemaside A1 and Compound K likely target effectors downstream of PDK1. The inhibition of the PDK1 phosphorylation by Arctigenin was expected because it targets PI3K (Figure 4A), which phosphorylates PDK1.

### Examining the effects of the screened compounds on the Akt membrane migration

**Figure 6:**
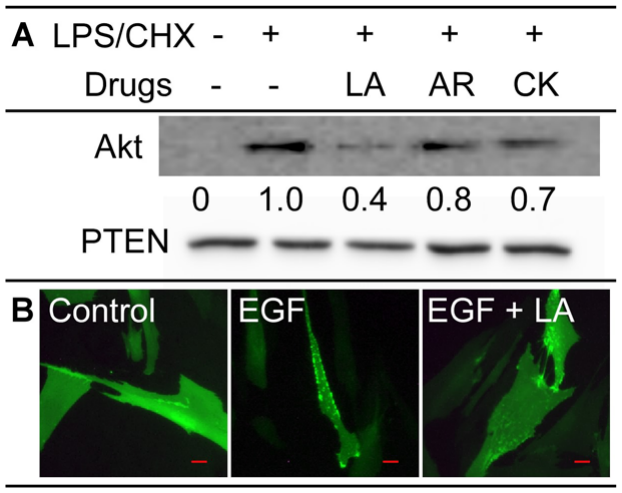
Determining the effects of the screened compounds on membrane localization of Akt. (**A**) Tat-expressing CHME5 cells were treated with 20 µM of the different drugs and LPS/CHX before having compartmental protein extraction done. Membrane fractions were then analyzed by western blot analysis for total Akt level. PTEN was used as an internal loading control, since it is present on the membrane and its level does not change under different treatment conditions (Figure 4B). (**B**) Human lung fibroblasts were transduced with Adenoviral vectors containing eGFP (control) or PH-Akt-eGFP and were treated with epithelial growth factor (EGF). Control cell picture (control) is eGFP. EGF picture is Akt-PH-eGFP expressing cells. EGF + LA picture is Akt-PH-eGFP expressing cells treated with Lancemaside A1. Akt-PH-eGFP localization to the plasma membrane was determined using a fluorescence microscope. Red scale bar indicates 20 µm distance.

Akt is phosphorylated by PDK1 at the plasma membrane [30]. We have established that Arctigenin inhibits PI3K, thus blocking PDK1 phosphorylation of Akt. However, both Compound K and Lancemaside A1 may use different mechanism(s) to inhibit pAkt levels in the cell. We postulated that inhibiting the movement of Akt to the plasma membrane might be one potential mechanism to explain pAkt inhibition by these two drugs. We used two independent methods to address this. First, we extracted the membrane fraction using the compartmental protein extraction protocol on treated cells to determine the total amount of Akt by immunoblotting. As shown in Figure 6A, Lancemaside A1 reduced the level of Akt at the membrane, whereas Arctigenin and Compound K had less inhibition on Akt trafficking to the plasmid membrane. PTEN was used as a loading control, since it is located at the plasma membrane and none of the drugs influence PTEN protein levels in the cell (Figure 4B). To further examine Akt membrane localization, we transduced human lung fibroblast cells with Adenovirus expressing Akt-PH-eGFP fusion protein or eGFP protein only. As shown in Figure 6B, 24 h after transduction cells were treated with epithelial growth factor (EGF). Control cells (control picture), transduced with eGFP and treated with EGF, had only cellular localization of eGFP. Positive control cells (EGF picture) had punctate Akt-PH-eGFP at the plasma membrane, whereas cells treated with EGF and Lancemaside A1 (EGF + LA picture) showed only cellular distribution of Akt-PH-eGFP, similar to control cells. Since it is assumed that Lancemaside A1 does not affect the activity of PI3K, it appears to inhibit Akt movement to the plasma membrane through inhibition of the PH domain. We conclude from these two data sets that Lancemaside A1 distinctly inhibits the localization of Akt to the plasma membrane, whereas Arctigenin and Compound K have little to no effect on Akt movement to the plasma membrane.

### Evaluating the effects of the screened compounds on two Akt down stream signals: mTOR and Bad

**Figure 7:**
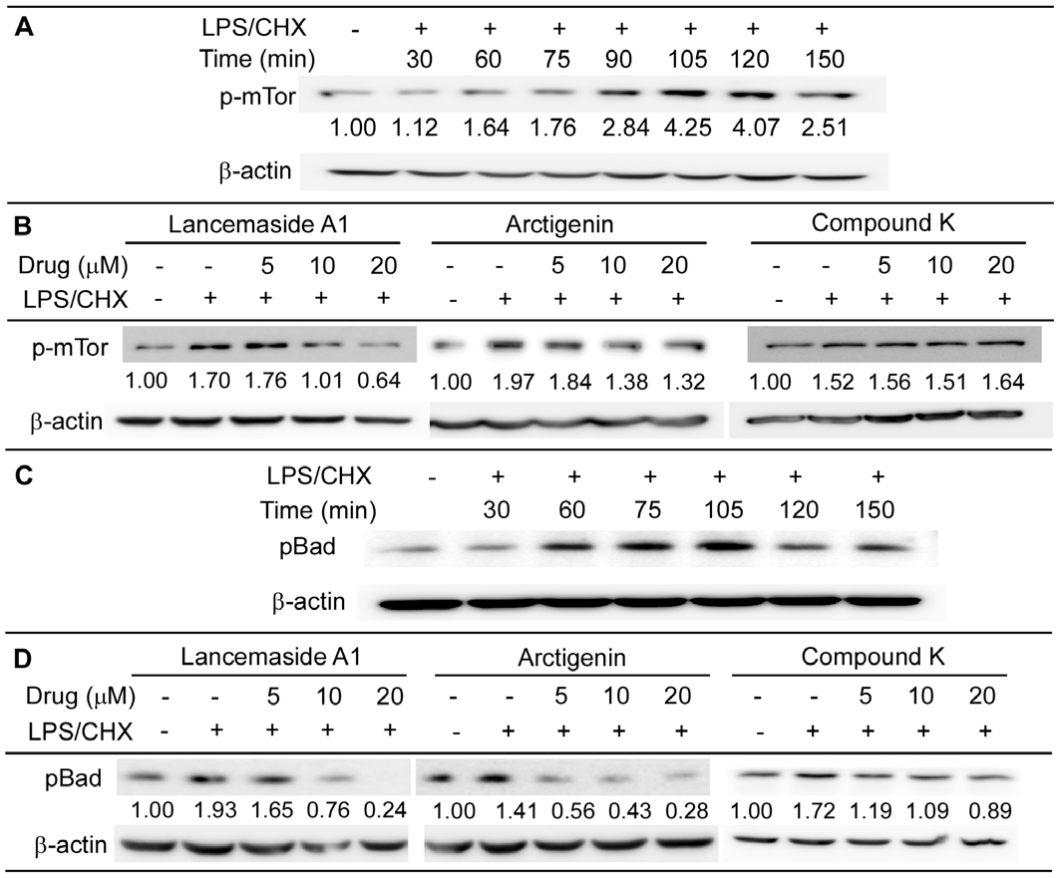
Evaluating the effects of the screened compounds on phosphorylation of mTOR and pBad. (**A**) A time course for mTor phosphorylation was done to optimize the assay. Maximum level of p-mTor was detected at 105 minutes after LPS/CHX treatment. (**B**) p-mTor level in Tat-expressing CHME5 cell lysates from different treatment conditions were examined at 105 minutes, and levels of p-mTor were normalized to β-actin. Averages of the data are shown below each blot. (**C**) Time course Bad phosphorylation was determined to optimize the assay, and maximum level of pBad was also detected at 105 minutes after LPS/CHX treatment. (**D**) pBad level in Tat-expressing CHME5 cell lysates from different treatment conditions were examined at 105 minutes, and levels of p-Bad were analyzed as described for mTor.

Next, we tested the influence of the screened compounds on two additional Akt downstream effectors, mammalian target of rapamycin (mTOR) and Bad. mTor has many cellular roles including regulation of cell growth, proliferation, survival, protein synthesis and metabolic homeostasis [31]. Bad appears to be involved in regulating metabolism and apoptosis [32]. As shown in Figure 7A and 7C, the LPS/CHX stress induced the phosphorylation of both mTOR (p-mTor) and Bad (pBad) in a time dependent manner, reaching maximum levels of phosphorylation at 105 minutes for both proteins. Cells treated with Lancemaside A1 and Arctigenin showed reduced levels of p-mTOR (Figure 7B) and pBAD (Figure 7D). Interestingly, Compound K treatment inhibited the level of pBad, but not p-mTOR. Possibly, the Akt inhibition mechanism of Compound K, which showed a relatively mild inhibitory effect on the upstream effectors of the PI3k/Akt pathway, appears to display differential downstream effects compared to the other two compounds.

## Discussion

Inhibition of the PI3K/Akt cell survival pathway is a highly attractive target for anti-cancer therapy. Many human cancer types display the activation of this pathway, enabling the cancer cells to survive and escape from anti-cancer responses. Importantly, several human onco-viruses, such as HPV, HTLV and HCV, also activate the PI3K/Akt pathway [1], [3], [4], [9]. The activation of the PI3K/Akt pathway has been considered as an important cellular event that viruses employ to ensure long-term and productive viral replication by extending the life span of the infected cells, and consequently leading to cell transformation. In addition, HIV-1 indirectly hijacks this pathway in macrophages by inhibiting the negative regulator of the pathway, PTEN, which extends the life span of the infected macrophages exposed to various virus-induced toxic stresses and contributes to the establishment of long-lived viral reservoirs [7], [8]. Thus, anti-PI3K/Akt agents could serve not only as effective anti-cancer drugs but also as anti-viral agents.

In this report, we employed the HIV-1 Tat-expressing CHME5 cell line, which exhibits an enhanced cell survival phenotype against LPS/CHX stress by a PI3K/Akt dependent manner, to screen previously identified anti-inflammatory and anti-stress compounds for anti-PI3K/Akt activity. Among those screened compounds, we identified three different compounds: Lancemaside A1, Arctigenin and Compound K, which antagonized the cytoprotective function of HIV-1 Tat in CHME5 cells during LPS/CHX stress (Figure 1B).

In our extensive and detailed mechanistic analyses, we found these three compounds to target different signals of the PI3K/Akt pathway. First, none of these compounds influence the level of PTEN, a negative regulator of the PI3K/Akt pathway. Second, Arctigenin is the only compound that affects the level of PIP3 and thus it likely targets PI3K. In fact, Arctigenin was previously reported as Akt inhibitor with potential anti-cancer effects [33]. This confirms that our Tat-expressing CHME5 model is effective at identifying anti-PI3K/Akt agents. Third, Akt phosphorylation requires the membrane migration of Akt. Lancemaside A1 clearly blocked the membrane migration of Akt as monitored by Akt-PH-GFP localization and by western blot analysis of membrane fractionation for Akt (Figure 6). Thus, these data directly support the hypothesis that Lancemaside A1 inhibits the PI3K/Akt pathway by targeting the membrane migration of Akt. Lastly, Compound K showed relatively weak signaling responses, compared to Lancemaside A1 and Arctigenin. However unlike Arctigenin and Lancemaside A1, Compound K did inhibit Akt activity, yet without reducing PI3K activity or blocking Akt membrane migration. Thus, these data support the idea that Compound K may directly inhibit Akt activity. More interestingly, when we investigated the downstream effectors of Akt, Lancemaside A1 and Arctigenin showed inhibitory effects against phosphorylation of mTOR, GSK3β and Bad. However, Compound K consistently and significantly reduced phosphorylation of GSK3β and Bad. Possibly, Compound K may target Akt kinase activity only for a few substrates, whereas Lancemaside A1 and Arctigenin may universally inhibit the downstream signals of the PI3K/Akt pathway. These mechanistic studies clearly support that the Tat-expressing CHME5 model system was able to screen anti-PI3K/Akt pathway molecules targeting diverse mechanistic steps of this pathway.

Recently Lancemaside A has been reported to control blood testosterone level in mice [34], as well as inhibiting LPS-induced inflammation [35]. Compound K has previously been identified as a putative anti-cancer agent [25], [36]. It can regulate metabolism via AMPK activation [37]. In addition, it has shown to regulate p38 and Akt [38], but has also been studied for its affects as an antidepressant [39]. Arctigenin has also been identified as an anti-cancer agent. It has the ability to inhibit STAT3 signaling pathway [40]. Recently, Arctigenin has been shown to inhibit IL-2 and interferon gene expression in T cells [41]. Collectively, these data suggest that all three compounds identified as PI3K/Akt pathways inhibitors by our screen also have additional effects on cells. However, our data suggest that Tat-induced cytoprotective phenotype of CHME5 cells is an effective screening system for agents targeting the PI3K/Akt pro-survival phenotype, which is commonly observed in cancer cells and cell types infected by several human pathogens. We have further pursued using Compound K as an anti-cancer agent *in vivo* (manuscript in preparation). We are encouraged by our results and believe additional tests should be conducted using various cancer models and virus-infection models in order to confirm the efficacy of the screened compounds as anti-cancer and anti-viral agents.

## Supporting Information

Data S1 **Live/Dead staining of Tat-expressing CHME5 cells.** Tat-expressing CHME5 cells were treated with and without 50 µg/ml LPS and 10 µg/ml cycloheximide for 24 hours in the presence of 10 µM Lancemaside A1, Arctigenin or Compound K. The Live/Dead assay (Invitrogen) stains dead cells with ethidium homodimer (red) and live cells with calcein (green). Images were captured using a fluorescent microscope. Images were manually counted to determine the numbers of live and dead cells.
(TIF)

Data S2 **Cell death for CHME5 control cells at different concentrations of drugs.** The Live/Dead assay was done, as described in Data S1, for the three different drugs at 5, 10 and 20 µM. The percentage cell death was plotted as mean and SEM for the three compounds.
(TIF)

Data S3 **PIP3 standard curve and data.** (**A**) Western dot blot analysis was done to generate a standard curve using 0.5, 1, 2, 4 and 5 pmoles of PIP3. Data were plotted to find the slope and y-intercept. (**B**) One representative data set is shown for lysates of Tat-expressing CHME5 cells treated under conditions. LPS/CHX treatment was 50 µg/ml LPS and 10 µg/ml cycloheximide. Lancemaside A1 (LA), Arctigenin (AR), Compound K (CK) and Wortmannin (WM).
(TIF)

Table S1 **Western blots were analyzed.** Data was normalized to either total proteins (Akt and GSK-3β) or β-actin (p-mTor, pBad, p-PDK1 and PTEN). Data of means and standard deviations from the western blot analysis are shown. Control lanes were set to 1.00. LPS is the positive control. Changes in intensities are shown for the different drug treatment groups.
(TIF)
