# Supplementary figures and images for: Novel PI3K/Akt Inhibitors Screened by the Cytoprotective Function of Human Immunodeficiency Virus Type 1 Tat
Source: PLoS One. 2011 Jul 12;6(7):e21781. doi: 10.1371/journal.pone.0021781 (PMC3134463; doi:10.1371/journal.pone.0021781)

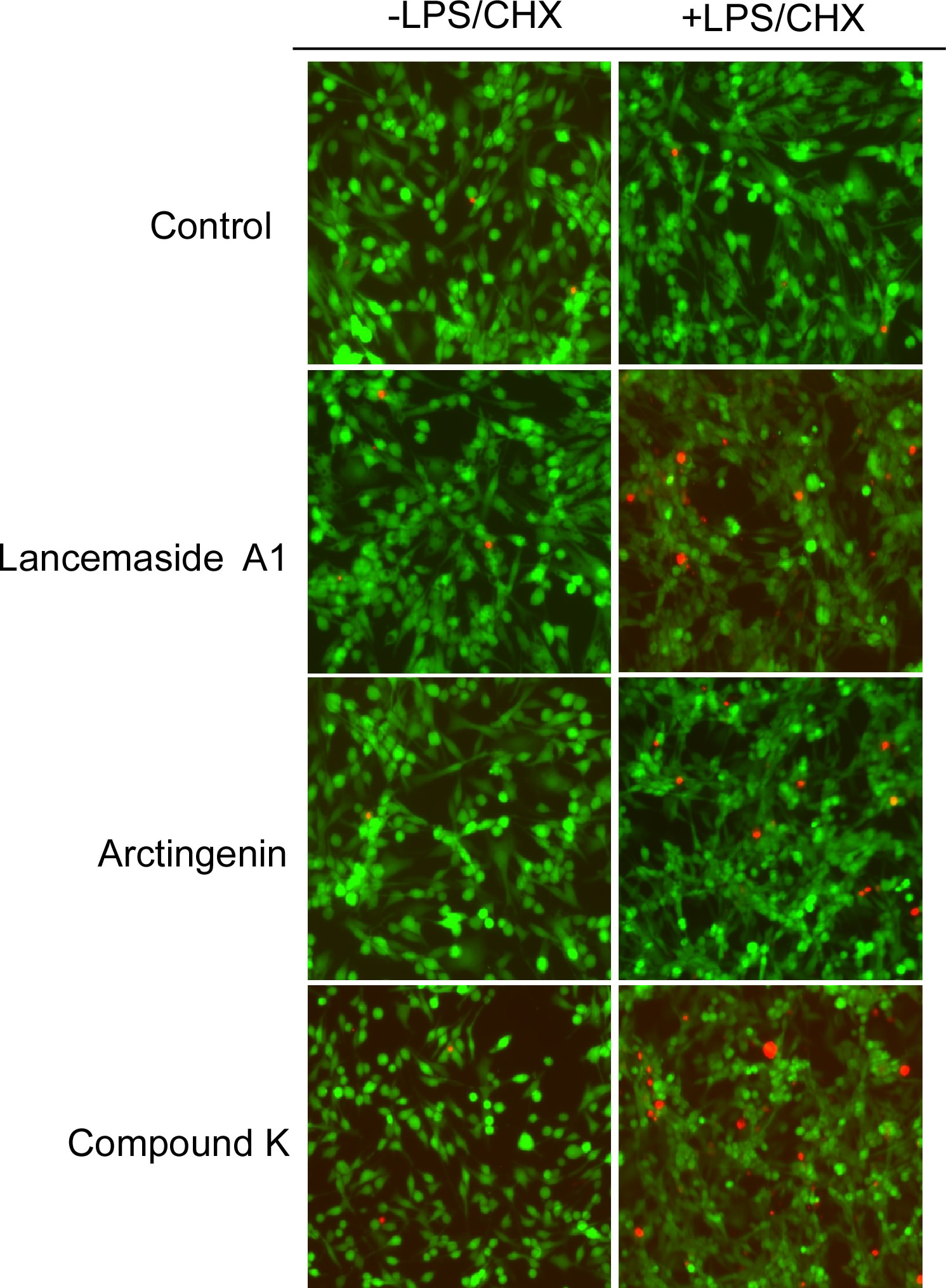

Supplement: Data S1 — Live/Dead staining of Tat-expressing CHME5 cells. Tat-expressing CHME5 cells were treated with and without 50 µg/ml LPS and 10 µg/ml cycloheximide for 24 hours in the presence of 10 µM Lancemaside A1, Arctigenin or Compound K. The Live/Dead assay (Invitrogen) stains dead cells with ethidium homodimer (red) and live cells with calcein (green). Images were captured using a fluorescent microscope. Images were manually counted to determine the numbers of live and dead cells. (TIF) [file pone.0021781.s001.tif]

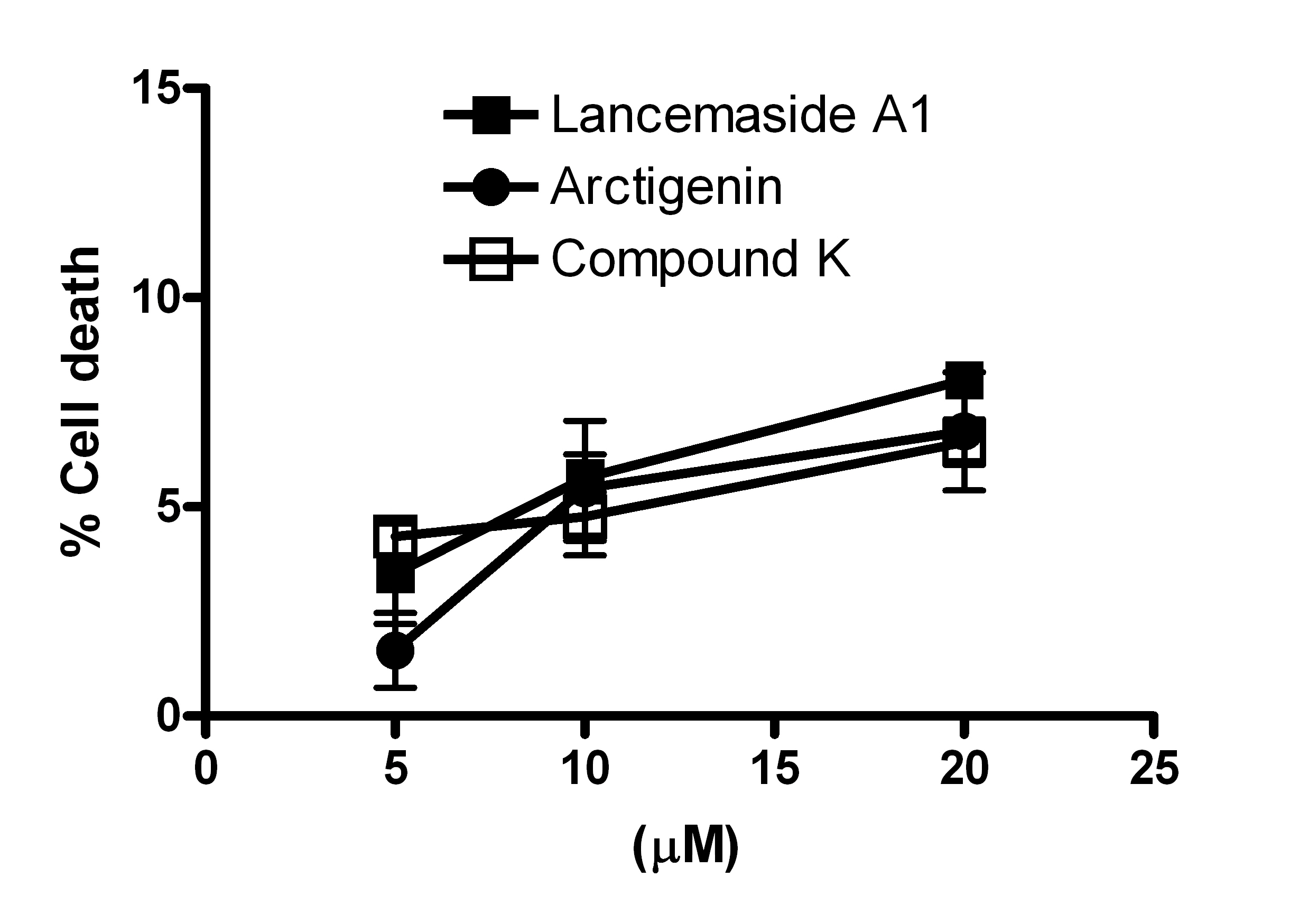

Supplement: Data S2 — Cell death for CHME5 control cells at different concentrations of drugs. The Live/Dead assay was done, as described in Data S1, for the three different drugs at 5, 10 and 20 µM. The percentage cell death was plotted as mean and SEM for the three compounds. (TIF) [file pone.0021781.s002.tif]

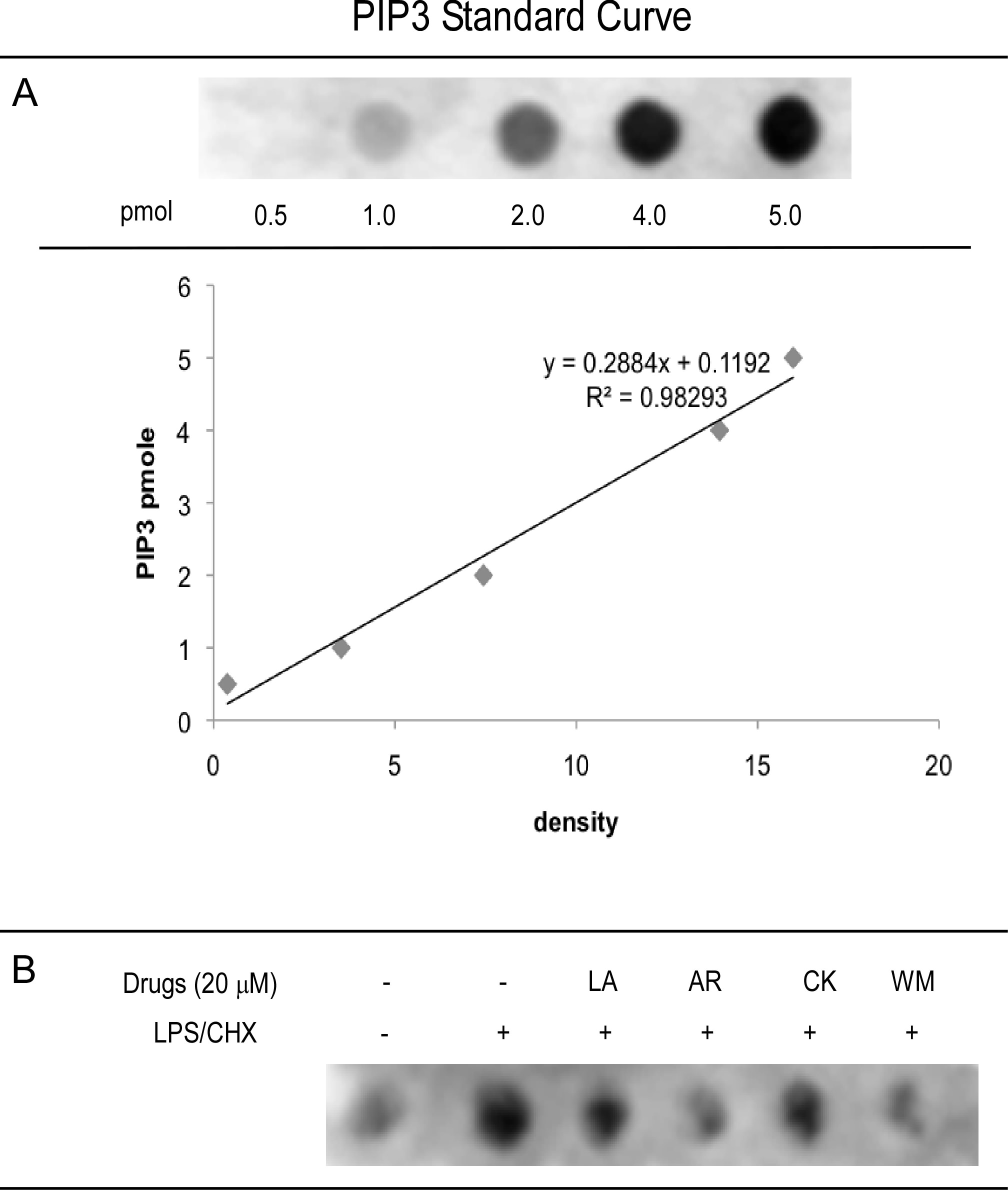

Supplement: Data S3 — PIP3 standard curve and data. (A) Western dot blot analysis was done to generate a standard curve using 0.5, 1, 2, 4 and 5 pmoles of PIP3. Data were plotted to find the slope and y-intercept. (B) One representative data set is shown for lysates of Tat-expressing CHME5 cells treated under conditions. LPS/CHX treatment was 50 µg/ml LPS and 10 µg/ml cycloheximide. Lancemaside A1 (LA), Arctigenin (AR), Compound K (CK) and Wortmannin (WM). (TIF) [file pone.0021781.s003.tif]

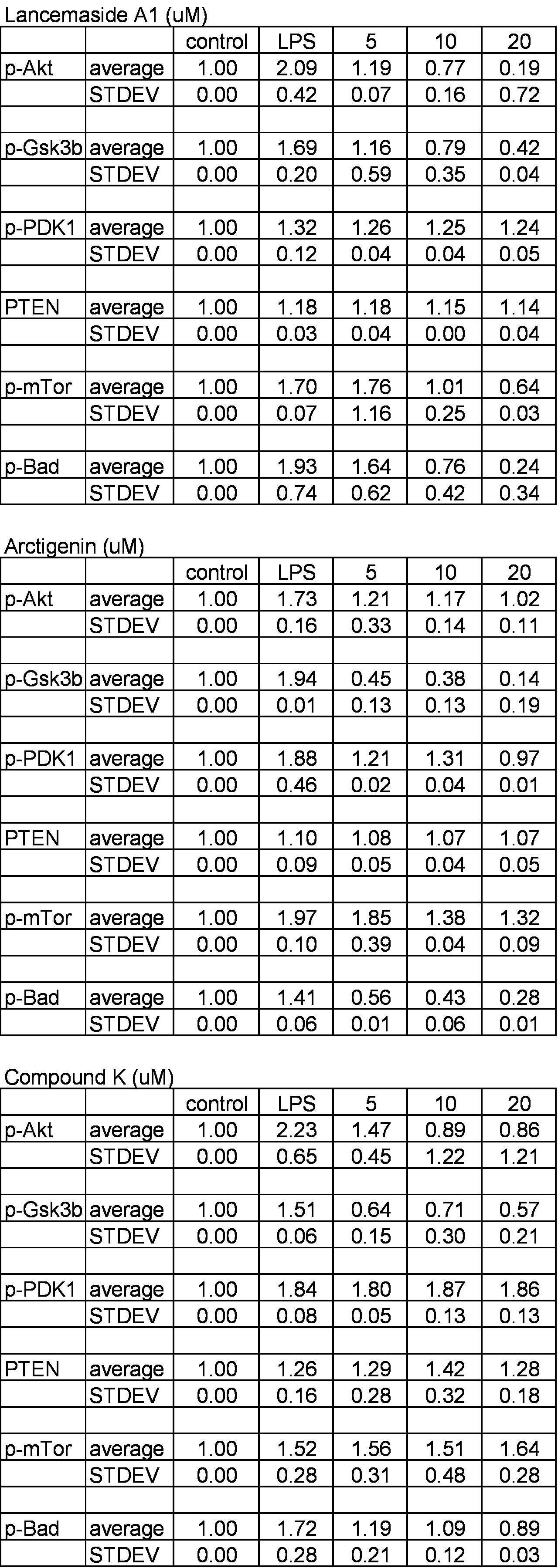

Supplement: Table S1 — Western blots were analyzed. Data was normalized to either total proteins (Akt and GSK-3β) or β-actin (p-mTor, pBad, p-PDK1 and PTEN). Data of means and standard deviations from the western blot analysis are shown. Control lanes were set to 1.00. LPS is the positive control. Changes in intensities are shown for the different drug treatment groups. (TIF) [file pone.0021781.s004.tif]
